# Role of Plant-Derived Natural Compounds in Experimental Autoimmune Encephalomyelitis: A Review of the Treatment Potential and Development Strategy

**DOI:** 10.3389/fphar.2021.639651

**Published:** 2021-06-28

**Authors:** Yu-Xin Guo, Yuan Zhang, Yu-Han Gao, Si-Ying Deng, Li-Mei Wang, Cui-Qin Li, Xing Li

**Affiliations:** ^1^ National Engineering Laboratory for Resource Development of Endangered Crude Drugs in Northwest China, The Key Laboratory of Medicinal Resources and Natural Pharmaceutical Chemistry, The Ministry of Education, College of Life Sciences, Shaanxi Normal University, Xi’an, China; ^2^ Department of Neurology, The First Affiliated Hospital of Zhengzhou University, Zhengzhou, China

**Keywords:** multiple sclerosis, anti-inflammatory, BBB, neuroprotective, nature product

## Abstract

Multiple sclerosis (MS) is an autoimmune disease of the central nervous system that is mainly mediated by pathological T-cells. Experimental autoimmune encephalomyelitis (EAE) is a well-known animal model of MS that is used to study the underlying mechanism and offers a theoretical basis for developing a novel therapy for MS. Good therapeutic effects have been observed after the administration of natural compounds and their derivatives as treatments for EAE. However, there has been a severe lag in the research and development of drug mechanisms related to MS. This review examines natural products that have the potential to effectively treat MS. The relevant data were consulted in order to elucidate the regulated mechanisms acting upon EAE by the flavonoids, glycosides, and triterpenoids derived from natural products. In addition, novel technologies such as network pharmacology, molecular docking, and high-throughput screening have been gradually applied in natural product development. The information provided herein can help improve targeting and timeliness for determining the specific mechanisms involved in natural medicine treatment and lay a foundation for further study.

## Introduction

Multiple sclerosis (MS) is an autoimmune disease that occurs in the central nervous system (CNS) (Alroughani et al., 2020). Experimental autoimmune encephalomyelitis (EAE) is an animal model used to study the pathogenesis or treatment of MS (Orefice et al., 2020). T-cell-mediated inflammation at the cellular level occurs in EAE and involves abundant inflammatory cell infiltration into the CNS when the blood-brain barrier (BBB) is damaged (Bagnoud et al., 2020). Also, the death of oligodendrocytes and the lack of neurotrophic factor production lead to further deterioration of the patient and disease advancement (Li et al., 2016).

The therapeutic effects of natural compounds have long been known (Maior and Dobrotă, 2013). Nearly 30% of the pharmaceuticals developed over the past 20 years have been derivatives of natural compounds (Patridge et al., 2016). For instance, the plant-derived compound artemisinin has been widely used in the treatment of malaria. In 2015, Youyou Tu was awarded the Nobel Prize in Physiology or Medicine for her contribution to this antimalarial drug (Di Nardo and Gilardi, 2020).

Traditional Chinese medicine (TCM) includes plants, animals, fungi, and minerals, with plants accounting for the largest proportion of therapeutic agents (Wang et al., 2014). There have been many studies of the treatment of EAE by TCM monomers containing flavonoids, phenol, and glycosides, among others (Ciumărnean et al., 2020; Ikram et al., 2020; Yang L. et al., 2020). Moreover, many researchers have studied the effect of chemical monomers by molecular docking, high-throughput screening, and network pharmacology (Chen et al., 2020; Liu J. et al., 2020; Xu et al., 2020a). Over the past 8 years, glycosides have been the most commonly studied compounds, and have been examined and developed with the intent of using them as a potential treatment for EAE (Giacoppo et al., 2013; Yin et al., 2014; Haghmorad et al., 2017; Madhu et al., 2019; Yang L. et al., 2020). Additionally, many studies of EAE and flavonoids have been conducted during the last 5 years (Chen et al., 2017; Xie et al., 2018; Cree et al., 2020a). By contrast, there have been few studies on phenols and triterpenes and their potential for EAE treatment. Therapeutic studies of EAE mainly concentrate on identifying traditional TCM monomers that have the ability to protect BBB integrity and possess anti-inflammatory or neuroprotective properties. Indeed, natural products have considerable therapeutic potential to ameliorate EAE for the treatment of MS (Cree et al., 2020b). At the cellular level, the deep mechanisms of TCM monomers that act during the treatment of diseases have been explored. However, the mechanism used by TCM monomers during the treatment of EAE is still not clear. The purpose of this review is to provide a theoretical basis and potential targets for natural small molecule compounds that can successfully treat EAE.

## Nature Products

### Flavonoid

#### Kurarinone

Kurarinone is a flavonoid that is extracted from the roots of shrubby sophora (*Sophora flavescens*) and is used to treat fever, acute dysentery, gastrointestinal hemorrhage, and eczema (Yang et al., 2018). Ethyl acetate is used to isolate kurarinone from *S. flavescens* roots (Yamahara et al., 1990). It has been reported that kurarinone can inhibit the development of tumors via promoting pathological cell apoptosis (Chung et al., 2019). Kurarinone is also an anti-inflammatory agent (Nishikawa et al., 2020) that plays an essential role in the immune regulation of Th1/Th17/Th2 when the i.p. injection dose is 100 mg/kg, which leads to a balance between pro-inflammatory cells and anti-inflammatory cells in the EAE model (Xie et al., 2018).

#### Naringenin

Naringenin is rich in fruits and can be extracted from dried navel orange (*Citrus sinensis*) peel powder by soaking it in 70% ethanol solution for 3 days (Ahmed et al., 2019). Several studies have shown that naringenin has a beneficial effect on Alzheimer’s disease, type 2 diabetes, and cancer (Aroui et al., 2020; Syed et al., 2020; Wu et al., 2020). Experiments have been conducted by adding 5% naringenin to the diet of mice or administering a therapeutic dose of 20–80 mg/kg naringenin by injection (Ahmad et al., 2014; Wang J. et al., 2018). Naringenin controls immunomodulatory functions, and it can regulate Tregs and balance the proportion of Th1/Th2, resulting in reduced inflammation in autoimmune arthritis (Ahmad et al., 2014). Moreover, naringenin inhibits the expression levels of transcription factors such as T-bet, PU.1, and RoR-γt that drive the differentiation of Th1, Th9, and Th17 and block the polarization of pathogenicity subsets of CD4^+^ T cells in the EAE model (Wang J. et al., 2018).

#### Hesperidin

The flavanone hesperidin is derived from citrus species, and it has demonstrated neuroprotective effects accompanied by reduced infiltration of leukocytes (Ciftci et al., 2015; Gandhi et al., 2020). The extraction agent referred to as a deep eutectic solvent is a green solvent that is effective for the extraction of hesperidin (Liu et al., 2019a). The therapeutic effect of hesperidin has been shown to be beneficial for neurodegenerative disorders such as Alzheimer’s disease, Parkinson’s disease, amyotrophic lateral sclerosis, and multiple sclerosis (Khan et al., 2020). In animal models, doses of hesperidin up to 50 mg/kg through subcutaneous injection resulted in obvious disease-relieving effects (Ciftci et al., 2015). Correspondingly, hesperidin can ameliorate clinical symptoms and suppress disease development *via* decreasing inflammatory factors, including tumor necrosis factor (TNF)-α and interleukin (IL)-1β, which results in a reduction of inflammation in the lesion sites in the EAE model (Ciftci et al., 2015). Hesperidin can adjust T cells to balance the ratio of pro-inflammatory and anti-inflammatory phenotypes, which is manifested by reduced expression of IL-6, IL-17, and TNF-α and subsequent enhanced levels of IL-10 and transforming growth factor (TGF)-β (Haghmorad et al., 2017).

#### Luteolin

Luteolin (Lut), a flavonoid obtained from various plants, has been shown to confer anti-inflammatory, anti-oxidative, and neuroprotective effects (Zhou W. et al., 2020). The methods for Lut extraction involve maceration, Soxhlet, reflux, ultrasound-assisted, enzyme-assisted, and supercritical fluid extraction (Manzoor et al., 2019). Lut is neuroprotective in various diseases including epilepsy, autism spectrum disorders, Alzheimer’s disease, Parkinson’s disease, traumatic brain injury, and MS (Nabavi et al., 2015a). Oral administration and intraperitoneal injection are the main administration routes for Lut for a number of disorder models (El-Deeb et al., 2019; Imran et al., 2019). The dosages of Lut range from 1.2 to 50 mg/kg, and *in vivo* studies have verified that it has a significant effect in alleviating various diseases such as cancer and MS (El-Deeb et al., 2019; Imran et al., 2019). Lut promotes ciliary neurotrophic factor (CNTF) expression and has the ability to increase cAMP and the total antioxidant capacity. Also, Lut can decrease TNF and IL-1 expression through the NF-κb signaling pathway in EAE (El-Deeb et al., 2019).

#### Icariin

Epimedium, commonly known as barrenwort or bishop’s hat (*Epimedium brevicornu* Maxim), is primarily used as a tonic, anti-rheumatic, and anti-cancer agent and is also is involved in neuroplasticity (Dietz et al., 2016; Tan et al., 2016). Epimedium A, B, and C, similar to icariin (ICA), are beneficial for osteoporosis and confer immunoregulatory effects (Meng et al., 2005). They are obtained by boiling extraction and Soxhlet extraction, as well as a new method known as ultrasound-assisted extraction (Zhang et al., 2008). Epimedium flavonoids, primarily containing ICA and epimedin A, regulate cells and inflammatory response to relieve the symptoms of EAE via a host of mechanisms such as reducing the level of Iba-1 and GFAP, which indicate reduced astrogliosis and decreased production of various inflammatory factors (Yin et al., 2012). Up to 300 mg/kg of ICA can be delivered to mice by gavage and at high doses (Wei et al., 2016). ICA relieved the inflammation of EAE induced by the MOG_35-55_ peptide (Wei et al., 2016). In addition, ICA alleviated the severity of relapsing-remitting EAE induced by PLP_139–151_ *via* the inhibition of microglial activation (Cong et al., 2020). ICA also decreases the number of Th17 and Th1 cells and protects the BBB (Shen et al., 2015).

#### Baicalin

Chinese/Baikal skullcap (*Scutellaria baicalensis* Georgi) contains flavonoids, terpenoids, and glycosides with anti-cancer, anti-oxidative, and anti-inflammatory effects (Shen et al., 2021). Baicalin (Ba) and aglycon baicalin are the principal flavonoid derivatives obtained from the roots of *S. baicalensis*, and they possess structural similarities (de Oliveira et al., 2015). Ba, with anti-inflammatory and immunomodulatory properties, plays a tremendous role in neuroinflammatory diseases (Li et al., 2020a). At present, the latest technology used to extract baicalin is the deep eutectic solvent ultra-high pressure method (Hao et al., 2020). The study has shown that intraperitoneal administration of Ba at a dose of 100 mg/kg can effectively alleviate the development of EAE in mice (Zhang et al., 2015). There is additional evidence that Ba inhibits the development of Th17 cells *via* promoting the expression of SOCS3 and reducing the production of pro-inflammatory factors IFN-γ and IL-17, leading to amelioration of EAE severity (Zhang et al., 2015; Li et al., 2020b).

#### Eriodictyol

Eriodictyol (EDT) is a flavonoid that is obtained from various fruits and possesses several bioactive activities, including anti-inflammatory, neuronal protection, and anti-oxidation (He et al., 2018; Habtemariam, 2019). EDT is usually extracted by ultrasound-assisted methods (Chotphruethipong et al., 2019). EDT has demonstrated a wide range of therapeutic effects, with an apparent pharmacological effect at doses from 0.25 to 100 mg/kg (He et al., 2018; Islam et al., 2020; Yang T. et al., 2020), and intraperitoneal injection is the primary mode of administration (He et al., 2018; Islam et al., 2020). Specifically, the anti-inflammatory effect of EDT is achieved via multiple signaling pathways, such as p38 mitogen-activated protein kinases (MAPK), Jun-N terminal kinase (JNK), and cyclooxygenase (COX)-2 (Lee et al., 2013). In addition, EDT inhibits the development of EAE by decreasing the polarization of Th17 and Th1 cells and increasing the number of Treg cells (Yang T. et al., 2020). Further research showed that EDT directly entered into the binding pocket of ROR-γt and prompted a conformational alteration that led to the suppression of the receptor's activity (Yang T. et al., 2020).

#### Quercetin

Quercetin, a flavonoid found in apple (*Malus domestica*) peel and vegetables, is obtained by ultrasonic-assisted extraction and the application of natural deep eutectic solvents (Vasantha Rupasinghe et al., 2011; Wei et al., 2020). Quercetin possesses anti-inflammatory, antioxidant, and neuroprotective properties (Costa et al., 2016; Marunaka et al., 2017; Xu et al., 2019). Besides, it can increase the survival rate of neural precursor cells (Ichwan et al., 2021). In a variety of mouse animal models, quercetin has shown prominent immunomodulatory activity. For example, based on a dose of 50 mg/kg daily i.p., quercetin significantly reduced clinical scores and prevented leukocyte infiltration in mice with acute EAE (Hendriks et al., 2004). It had been previously shown that quercetin exhibits inflammatory, inhibitory, and demyelinating blockade functions in EAE mice, following treatment with 2.5 or 5 mg/kg (Muthian, 2004). After analysis, it was determined that it alleviates the disease by blocking Th1 differentiation (Muthian, 2004).

### Glycoside

#### Glucosinolates

Glucosinolates can be hydrolyzed as sulforaphane, which is widely used to treat acute and chronic neurodegenerative diseases (Tarozzi et al., 2013). The usual dose is 10 mg/kg administered intraperitoneally (Foti Cuzzola et al., 2013; Galuppo et al., 2013; Giacoppo et al., 2013). A practical method to extract glucosinolates consists of grinding seed material and adding it to columns with petroleum ether and 10.8-fold water to extract the effective ingredients and then using 70% ethanol precipitation to separate the glucosinolates (Chen et al., 2019). Glucosinolates, which are obtained from Brassicaceae, can relieve inflammatory response and regulate various inflammatory factors. By significantly preventing the loss of axons, demyelination, and neurodegeneration *via* regulating the signaling pathways of NF-κB and IkB-α, glucosinolates slow the progression of EAE (Giacoppo et al., 2013).

#### Ginsenoside

Ginsenosides are extracted from Asian ginseng (*Panax ginseng*) and notoginseng (*Panax notoginseng*), which are commonly consumed as herbs, functional food, and health supplements (Zhu et al., 2014; Piao et al., 2020; Sharma and Lee, 2020). Several novel widely used technologies for ginsenoside extraction include a deep eutectic solvent-salt aqueous two-phase system, microwave-assisted extraction, ultra-high-pressure, and aqueous ionic liquid-based ultrasonic methods (Zhang et al., 2006; Liang et al., 2019; Zhao et al., 2019). Different types of ginsenosides have various pharmacological effects. Administration methods include gavage, tail vein injection, and intraperitoneal injection (Li X. et al., 2020). The doses used in various animal experiments range from 5 to 400 mg/kg (Li X. et al., 2020). With multiple pharmacological activities, ginsenoside Rd possesses anti-inflammatory, antioxidative, antiapoptotic, and neuroprotective abilities. It decreases the differentiation of Th1 cells, increases the polarization of Th2 cells, promotes trophic factor production, and protects BBB integrity, resulting in amelioration of EAE development (Zhu et al., 2014; Nabavi et al., 2015b). Moreover, ginsenoside Rg1 can prevent and treat inflammatory disease (Li X. et al., 2020), and ginsenoside Rh2 possesses anticancer properties (Li X. et al., 2020).

#### Astragaloside IV

Astragaloside IV (ASI) is abundant in astragalus/milkvetch (*Astragalus membranaceus* (Fisch.) Bunge) (He et al., 2013; Wang et al., 2017). The extraction method for ASI is ultrasonic-assisted liquid extraction (Qin et al., 2011). Doses of ASI ranging from 25 to 50 mg/kg have produced markedly therapeutic pharmacological effects (Wang et al., 2017; Yang L. et al., 2020). ASI is administered intraperitoneally (Wang et al., 2017), and anti-inflammatory properties that benefit diabetes treatment have been observed (Xie and Du, 2011; Tan et al., 2020; Zhou X. et al., 2020). In addition, in the EAE model, ASI inhibits the differentiation and maturation of dendritic cells by inhibiting CD11c, CD86, CD40, and MHC II activation. At the molecular level, ASI reduces the RNA expression levels of cytokines IL-6, IL-12p35, and IL-12p40 by regulating the NF-κB signaling pathway (Yang L. et al., 2020).

#### Paeoniflorin

Paeoniflorin (PF), which is derived from Chinese peony (*Paeonia lactiflora*), has demonstrated effective anti-inflammatory regulation of rheumatoid arthritis and systemic lupus erythematosus (Tu et al., 2019). The processes used to extract PF include ultrasonic and reflux extraction (Ji et al., 2020). PF also exhibits positive actions on liver cancer *via* hepatic, cholestatic, and liver fiber attenuation, and prevents nonalcoholic fatty liver disease (Ma X. et al., 2020). PF is administered orally, intraperitoneally, and intravenously, and specific pharmacological effects have been observed for the dose range of 5–200 mg/kg (Zhang et al., 2017; Ma X. et al., 2020; Zhou Y. et al., 2020). PF regulates the activity of B lymphocytes, T cells, and dendritic cells (DCs) and decreases IL-1, TNF-α, IL-17, and IFN-γ expression (Baldwin, 2001; Zhou Y. et al., 2020). It also induces activation of the NF-κB and mitogen-activated protein kinase (MAPK) signaling pathways and thus confers anti-inflammatory and immunoregulatory effects (Baldwin, 2001; Zhou Y. et al., 2020). Also, PF efficiently blocks the activation of pro-inflammatory cells and balances pro-inflammation and regulatory cells in various inflammatory diseases (Zhang and Wei, 2020). Similarly, PF inhibits the progression of EAE by decreasing Th17 cell polarization and DC cell activation, which can be induced by IKK/NF-κB and JNK (Zhang et al., 2017).

#### Anemoside A3

In mouse models, the mice were dosed with anemoside (AA3) at 30–300 mg/kg (Ip et al., 2015; Ip et al., 2017; Wang C. et al., 2019). AA3 is usually administered intraperitoneally and orally (Ip et al., 2015; Ip et al., 2017). AA3 is the primary effective component from pulsatilla (*Pulsatilla chinensis*), and it offers neuroprotection and (Ip et al., 2015) inhibits inflammation via modulation of toll-like receptor 4 (TLR4)/myeloid differential protein-88 (MyD88) (He et al., 2020). In addition, AA3 reduced the infiltration of inflammatory cells, regulated Th1 and Th17 cells, and decreased the expression of transcription factors STAT4 and STAT3 in the EAE model (Ip et al., 2017). AA3, with anti-tumor, neuroprotective, and immunomodulatory effects, can be regarded as a possible drug for the treatment of neurodegenerative and autoimmune diseases (Yoo and Park, 2012; Li et al., 2020c).

### Triterpenoid

#### Ursolic Acid

Ursolic acid (UA) is a triterpenoid that plays an important role in neurodegenerative disease (Yoo and Park, 2012). The sources of UA are extensive, and it can be extracted from plants, fruits, and vegetables (Khwaza et al., 2020). Ultrasonic extraction, microwave extraction, and supercritical fluid extraction are the primary techniques used for UA extraction (Xia et al., 2011; Alves Monteath et al., 2017; López-Hortas et al., 2018). Furthermore, conventional maceration, Soxhlet extraction, and heat reflux extraction can be applied to extract UA. At present, UA is known to have various pharmacological effects such as anti-inflammatory, anti-cancer and anti-oxidation (Mlala et al., 2019). UA treatment at 5–150 mg/kg is usually given to rats by gavage or intraperitoneally (Xu et al., 2011; Shin et al., 2012; López-Hortas et al., 2018; Zhang Y. et al., 2020). In terms of the differentiation of CD4^+^ T cells, UA suppresses the expression of pro-inflammatory cytokine IL-17, mainly through inhibiting the function of transcriptional factor ROR-γt, which results in the blockage of Th17 cell differentiation in EAE (Xu et al., 2011). Additionally, UA induced ciliary neurotrophic factor production in astrocytes through peroxisome proliferation activated receptor γ (PPARγ)/CREB signaling and enhanced the level of myelin-related gene by activating PPARγ during the maturation of oligodendrocytes (OLG) (Zhang Y. et al., 2020).

#### Carnosol

Carnosol (CA) is a diterpene derived from rosemary (*Rosmarinus officinalis*) that possesses anti-oxidative and anti-inflammatory properties (de Oliveira, 2015). Supercritical fluid extraction, ultrasound, microwave, or deep eutectic solvents can be used to isolate CA (Jacotet-Navarro et al., 2015; Jakovljević et al., 2021; Lefebvre et al., 2021). Nicole et al. reported that CA attenuated dendritic cell glycolysis and spare respiratory capacity under the stimulation of lipopolysaccharide (LPS) (Campbell et al., 2019). Effects were noted in mice when intraperitoneal injection of CA was administered at doses of 10 and 50 mg/kg (Rodrigues et al., 2012; Li X. et al., 2018). Furthermore, CA displayed a significant therapeutic effect on active and passive EAE. CA decreased the differentiation of Th17 cells by suppressing signal transducer and activator of transcription 3 (STAT3) phosphorylation and blocking transcription factor NF-κB nuclear translocation. Also, CA switched the phenotypes of microglia, and it was observed that M1-type microglia transformed to M2-type (Li X. et al., 2018).

#### Cornel Iridoid Glycosides

Cornel iridoid glycoside (CIG), which is obtained from Japanese cornelian dogwood (*Cornus officinalis*), reduced inflammatory cell infiltration and expression of proinflammatory factors from pathogenic Th1 and Th17 cells in the EAE model (Yin et al., 2014). In addition, microglial cells are closely associated with inflammation and can affect the progression of MS (Pinto and Fernandes, 2020). CIG treatment markedly decreased the number of M1-type microglial cells, which are characterized by pro-inflammatory effects, and increased the number of M2-type microglial cells, which possess anti-inflammatory characteristics (Qu et al., 2019). CIG promotes brain-derived neurotrophic factor (BDNF) and nerve growth factor (NGF), which are neurotrophic factors that control survival, differentiation, and growth of neurons (Qu et al., 2016). Intragastric administration of CIG at 30–120 mg/kg resulted in significant therapeutic activity (Yin et al., 2014; Qu et al., 2019).

#### Glycyrrhizic Acid

Glycyrrhizic acid (GA), isolated from Chinese licorice (*Glycyrrhiza uralensis*), exhibits anti-viral, anti-bacterial (Wang et al., 2015), anti-inflammatory, and neuroprotective activities (Kao et al., 2014). Hot water extraction and microwave extraction are used to isolate GA (Sun et al., 2008; Shabkhiz et al., 2016). GA has been used to treat COVID-19 (Bailly et al., 2020) and liver disease (Li et al., 2019). And therapeutic effects have been observed when it is orally and intraperitoneally administered at a dose of 2–80 mg/kg (Liu et al., 2011; Akman et al., 2015). GA decreases the expression of high-mobility group box protein 1 (HMGB1), which subsequently ameliorates neuroinflammation in the EAE model (Li J. et al., 2018). This beneficial effect may be attributed to GA downregulating Iba1 expression and inhibiting microglial activation (Song et al., 2013; Zhou et al., 2015). Significantly, GA induces oligodendrocyte precursor cell (OPC) differentiation *via* regulation of the glycogen synthase kinase-3 (GSK-3β) signaling pathway and promotion of remyelination in EAE (Tian et al., 2020).

### Others

#### Matrine

Matrine (MAT), an alkaloid derived from *Sophora flavescens*, possesses multiple pharmacological activities, including anti-cancer (Cao and He, 2020), anti-inflammatory, and immunosuppressive (Oveissi et al., 2019). Molecularly imprinted solid-phase and ultrasound-assisted enzymatic methods are used to extract MAT (Guo et al., 2011; Wang H. et al., 2018), which has been used to treat Alzheimer’s disease, spinal cord injury, and rheumatoid arthritis (Zhang H. et al., 2020). MAT is injected intraperitoneally at 10–250 mg/kg, and within this range, the injected drugs may have corresponding pharmacological effects (Wang M. et al., 2019; Balkrishna et al., 2020; Sun N. et al., 2020). In the development of EAE, astrogliosis played a significant role (Hibbits et al., 2012; Moreno et al., 2013). Correspondingly, MAT inhibits astrogliosis by downregulating the expression of S1P, leading to alleviation of the severity of EAE (Ma W. et al., 2020). Otherwise, MAT can inhibit OLG apoptosis, resulting in decreased demyelination in the EAE model (Wang M. et al., 2019). Apart from this, Wang et al., also showed that MAT upregulated autophagy-related protein Beclin1 and enhanced mitochondrial autophagy, thereby alleviating demyelination (Wang M. et al., 2019).

#### Scopoletin

Scopoletin, a phenolic coumarin derived from various medical or edible plants, possesses various medical properties and exhibits anti-inflammatory, anti-hypotensive (Balkrishna et al., 2020), anti-diabetic (Choi et al., 2017), and anti-aging activities (Nam and Kim, 2015). Supercritical extraction and a new modern pressurized cyclic solid-liquid method are used for scopoletin extraction (Jokić et al., 2016; Zarrelli et al., 2019). Under the inflammatory condition of EAE, DCs and antigen-presenting cells play an important role in disease occurrence, which can activate T cells after antigen presentation (Zozulya et al., 2010). Intraperitoneal injection of scopoletin at 50 mg/kg decreases the expression of MHC class II, CD80, and CD86 costimulatory molecules and inhibits NF-κB phosphorylation (Banihani, 2018). Scopoletin also downregulates the pathogenic Th1/Th17 inflammatory cell response after the suppression of the activation of DCs, which alleviates EAE severity (Zhang et al., 2019a).

#### 6-Gingerol

6-Gingerol (6-Gin), the main active compound from ginger (*Zingiber officinale*)(Banihani, 2018), possesses anti-tumor and immunomodulatory properties (Chen et al., 2018; Liu et al., 2019b). The ultrasonic-assisted water method and subcritical water are used to extract 6-Gin (Syed Jaapar et al., 2017; Ko et al., 2019). Mice have been treated with 0.25–15 mg/kg 6-Gin, which is administered orally and intraperitoneally (Kawamoto et al., 2016; Han et al., 2019; Zhang et al., 2019b; Tsai et al., 2020). 6-Gin effectively inhibits the development of neurodegenerative diseases, such as Alzheimer’s disease (Halawany et al., 2017). It has been reported that 6-Gin reduces inflammatory response *via* the inhibition of T cell activity (Kawamoto et al., 2016). Additionally, 6-Gin suppresses lipopolysaccharide-induced DC activation and induces tolerogenic DCs. Furthermore, 6-Gin blocks the function of DCs by inhibiting the phosphorylation of NF-κB and mitogen-activated protein kinase (MAPK), therefore ameliorating the severity of inflammation in the CNS and reducing the progression of EAE (Han et al., 2019).

#### Ellagic Acid

Ellagic acid, a polyphenolic compound, is endowed with anti-tumor and anti-angiogenic activity, and it promotes humoral immunity (Zhao et al., 2013; Ceci et al., 2018). It can be extracted from various fruits and bacteria, such as pomegranate (*Punica granatum* L.) and strawberry (*Fragria ananassa* Duch.) (Ceci et al., 2018). EA can be extracted by ultrasound-assisted method (Zhang et al., 2010; Assunção et al., 2017). The therapeutic dose range for EA is 0.1–300 mg/kg, and it can be orally and intraperitoneally administered (Baradaran Rahimi et al., 2020). In the animal model of EAE, EA reduced inflammation, and blocked myelin loss and axonal damage (Kiasalari et al., 2021). EA also promotes neuroprotection by decreasing GFAP and Iba1 immunoreactivity (Busto et al., 2018; Kiasalari et al., 2021).

## Novel Strategies Used for Developing New Natural Products

### Network Pharmacology

**FIGURE 1:**
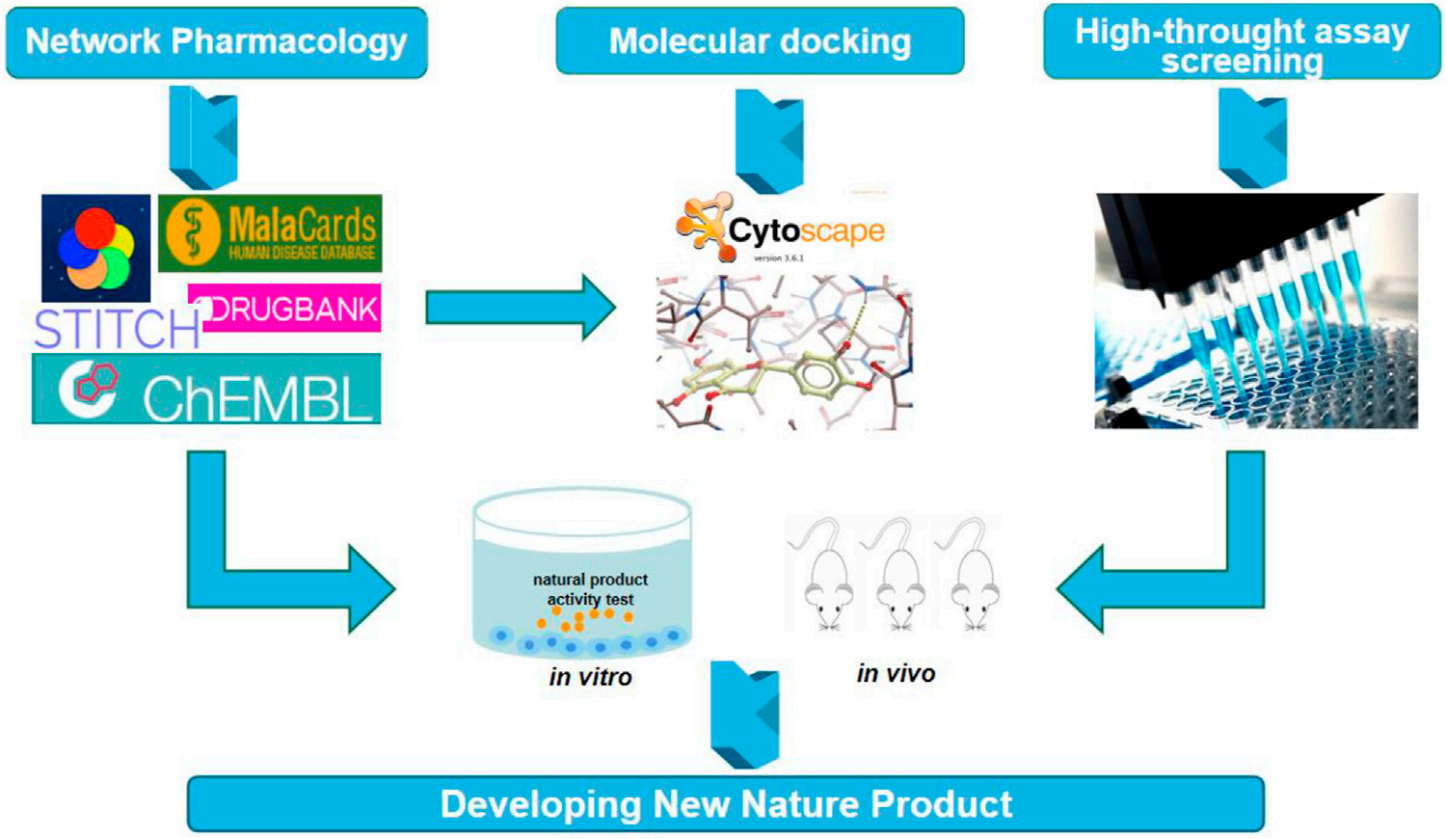
Novel strategies applied to developing nature product medicine.

The development of new valuable natural products is becoming more and more difficult, so new technologies need to be applied in this field. In this part of the content, we will briefly introduce the application of Network pharmacology, molecular docking, and high-throughput assay for screening technology in the field of natural medicine (Figure 1).

Network pharmacology is an interdisciplinary subject that integrates biological networks, analyzes the relationship between drugs and nodes or network modules, and accelerates the identification of drug targets and the discovery of new biomarkers (Kibble et al., 2015). Our understanding of the biological basis of TCM treatment can be attributed to network pharmacology (Wang et al., 2020)

A study was performed that used network pharmacology to research the molecular mechanisms of Lian Hua Qing Wen (a mix of 13 herbs) in novel coronavirus disease, and the results showed that its mechanisms are closely related to modulating inflammation, antiviral action, and protecting the lungs (Zheng et al., 2020). Network pharmacology was also applied in identifying the active compounds from Kai-Xin-San, which is a TCM that consists of 1) ginseng (*Panax ginseng*), 2) snakeroot (*Polygala tenuifolia* Wild.), 3) Shi-Chang-Pu (*Acorus tatarinowii* Schott), and 4) Poria mushroom (*Wolfiporia extensa* Ginns.). Additionally, network pharmacology was used to determine which genes correlated with Alzheimer’s disease, for the purpose of finding potential signaling pathways and novel compounds (Yi et al., 2020). Network pharmacology analysis was also used to find various active compounds for treating ulcerative colitis that contained formononetin, kushenol N, and kuraridin, to lay the foundation for studying the disease (Chen et al., 2020). Overall, the study of network pharmacology can preliminarily predict the component monomers and disease targets of TCM to lay a foundation for elucidating the therapeutic mechanism of TCM, and it also can be applied in the study of EAE.

Here, we have summarized multiple online databases of Chinese herbal medicines or small molecule drugs. NPASS (http://bidd2.nus.edu.sg/NPASS) integrates species sources of natural products and connects natural products to biological targets via experimental-derived quantitative activity data (Zeng et al., 2018). TCMSP (http://tcmspw.com/tcmsp.php) contains chemicals, target, and drug-target networks, and is a pharmacology platform for Chinese herbal medicines (Ru et al., 2014). DrugBank (http://www.drugbank.ca) combines chemical and pharmacological drug data with comprehensive drug targets, including sequence, structure, and pathway (Law et al., 2014). STITCH (http://stitch.embl.de/) is a database of compound-protein interactions and can also be used for compound target prediction (Szklarczyk et al., 2016). ChEMBL (http://www.ebi.ac.uk/chembldb) contains 6,900 compounds and provides structure, function, and compound targets (Mendez et al., 2019). Moreover, there are disease databases, such as DisGeNET (http://www.disgenet.org/), which is a comprehensive database of gene-disease associations (Pinero et al., 2020). The MalaCards (http://www.malacards.org/) database includes therapeutic compounds, disease categories, profiles, and related genes (Rappaport et al., 2017). For greater insight, the disease databases can be combined with the active compound target databases via genetic and protein sequences.

### Molecular Docking

Molecular docking is a drug design method based on the characteristics of receptors and the interaction mode between receptors and drug molecules. It is mainly a theoretical simulation method used to study the interaction between molecules (ligand and receptor), predict their binding ability and affinity, and verify the experimental results by assay (Liu J. et al., 2020).

Molecular docking analysis plays a significant role in predicting new drugs and medicinal repurposing (Chatterjee et al., 2020). Molecular docking can be widely used in the study of the interaction between various small molecule compounds and protein. A typical example is the use of molecular docking to promote the study of UA’s target molecules. Molecular docking revealed that UA could combine with caspase-3 protein and inhibit caspase-3 activity. Additionally, experiments *in vivo* and *in vitro* demonstrated that UA could block hepatocellular apoptosis and relieve liver injury via suppressing apoptotic caspase-3 protein (Morales Torres et al., 2020). Correspondingly, molecular docking has been used to identify a novel ligand of the aryl hydrocarbon receptor (Ahr), namely, garlic acid. Garlic acid regulates the increase in the number of Treg cells and the decrease in pro-inflammatory cytokines in EAE, which clarifies the mechanism used by garlic acid to block Ahr and subsequently achieve disease remission (Abdullah et al., 2019). Molecular docking involves the preliminary prediction of signaling pathways to treat the disease and experimental verification. For confirmation, molecular docking technology can be combined with network pharmacology to identify novel natural compounds in TCM. Cytospace and SwissDock (http://www.swissdock.ch/) can also be used to simulate the interaction between proteins and small molecule compounds (Grosdidier et al., 2011).

### High-Throughput Assay For Screening

High-throughput assay for screening (HTS) technology is based on molecular and cellular levels of the experimental method. It is a rapid, sensitive, and accurate method that is used to simultaneously test thousands of novel compounds from natural products (Xu et al., 2020b). Simply, it processes a large amount of information through HTS and finds valuable information from it.

HTS can be used to identify active components of natural products and also small molecular chemical compounds. Some effective flavonoids (Tian et al., 2019), and terpenoids (Jackson et al., 2013) have been identified using Selleckchem’s products, which are helpful in the treatment of diseases (Morales Torres et al., 2020). For example, procyanidin B2 (PCB2) is a natural flavonoid that is found in common foods, and it can activate PPARγ and induce M2 polarization in mouse macrophages that inhibit the activation of inflammation in the lung tissue of rats (Tian et al., 2019). Otherwise, in immune disease, pteryxin, a coumarin derivative, is found via this website, and it can inhibit the production of LPS-induced peritoneal macrophages in mice, with the potential to be used for the treatment of Alzheimer’s disease (Orhan et al., 2017). Therefore, we can use this website to build a dedicated compound library and efficiently identify effective compounds by HTS.

## Conclusions and Prospects

Flavonoids, glycosides, triterpenes, and other monomers of TCMs can alleviate EAE through different mechanisms of action, including suppressing inflammatory response, promoting neural protection, and protecting BBB integrity. These data can help formulate a specific theoretical basis for the natural-product treatment of MS diseases.

**FIGURE 2:**
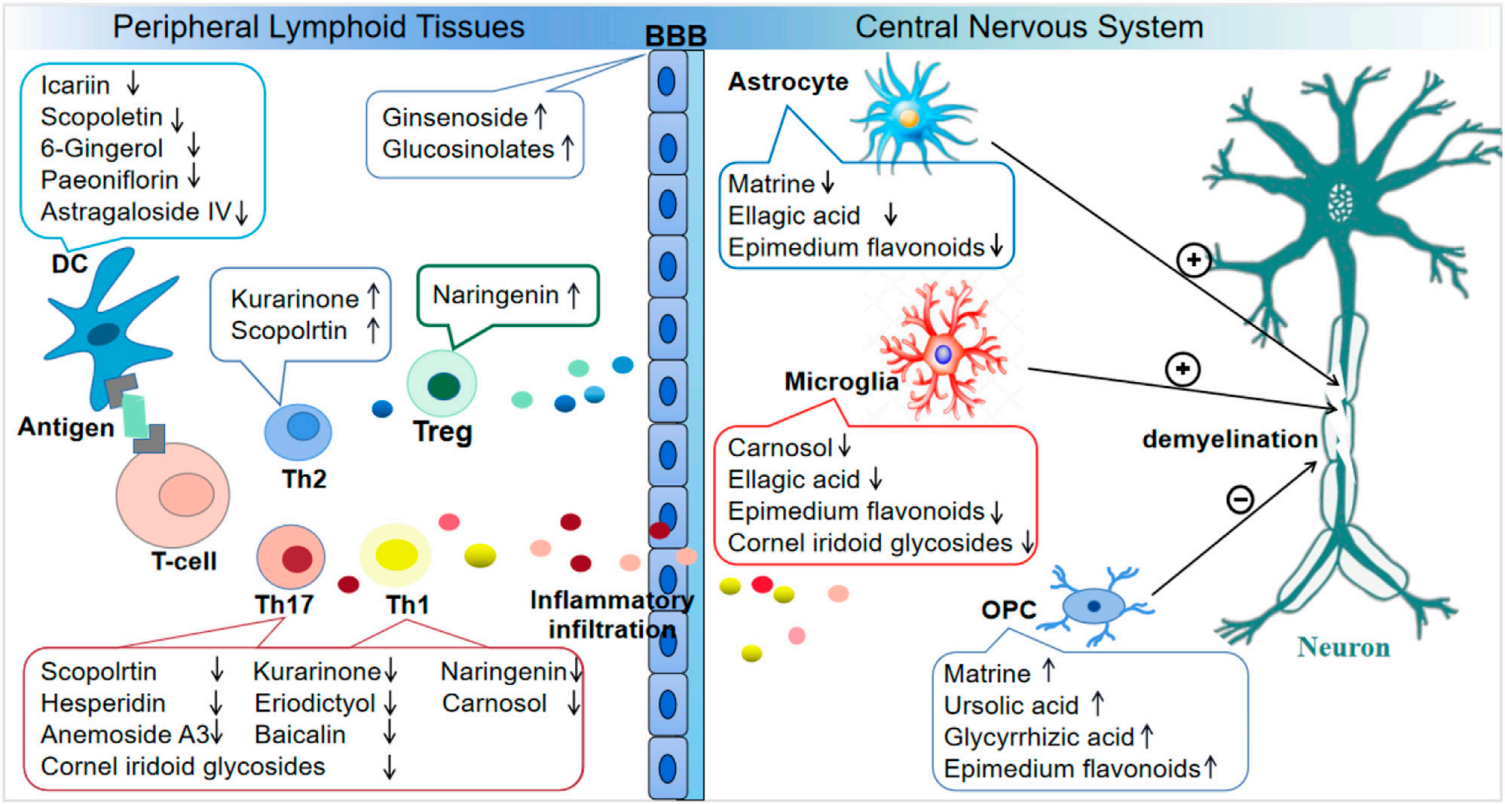
Different monomers of TCM act on various objects in EAE.

According to this review, many monomers in TCMs have a significant effect on EAE amelioration. In the EAE model, most of these Chinese herbal monomers can inhibit the production of inflammatory factors such as IL-1β and IL-17; promote anti-inflammatory factors such as IL-10, TGF-β, and others; and regulate pro-inflammatory and anti-inflammatory balance. Among them, ASI, PF, scopoletin, and 6-Gin can inhibit DC proliferation and differentiation. CA, CIG, and GA can promote the transformation of M1-type microglia into M2-type microglia and exert anti-inflammatory action. MAT counteracts inflammation by inhibiting astrocytes. In terms of BBB protection, glucosinolates and ginsenoside Rd can protect the BBB from damage, thereby reducing the severity of EAE. In addition, UA and GA promote OPC maturation and myelin regeneration. Therefore, monomer components derived from natural products have excellent prospects for the treatment of EAE, and finding monomers through the methods mentioned above represents the latest strategy. Overall, in EAE, different TCM monomers can act on various inflammatory cells or other related cells, including DCs, macrophages, T cells, microglia, and astrocytes. TCM monomers were also protective of neural cells and maintained BBB integrity. A summary of these agents is shown in Figure 2 and Table 1.

**TABLE 1:** Natural products act on the target cells of EAE.

	Compound	Structure	Sources	EAE model	Target cell	Effect	Ref
Flavonoid	Quercetin	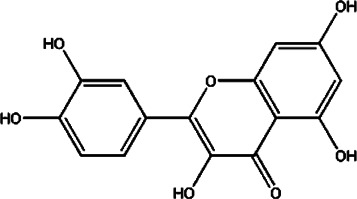	*Allium cepa L*	PP-EAE	Th1	Inhibit	Muthian and Bright (2004), Costa et al. (2016)
			*Asparagus officinalis L*				
			*Lactuca sativa L*				
	Baicalin	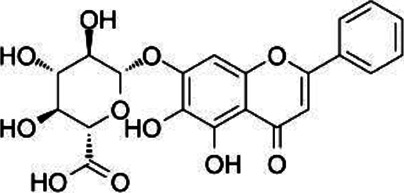	*Scutellaria baicalensis*	PP-EAE	Th17	Inhibit	Zhang et al. (2015)
					Th1		
	Icariin	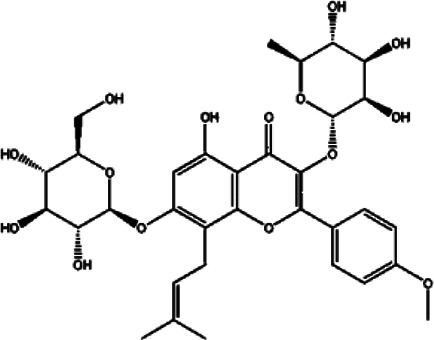	*Epimedium brevicornu*	PP-EAE	DC	Inhibit	Shen et al. (2015), Wei et al. (2016)
					Th1		
					Th17		
				RR-EAE	Microglia		
Flavonoid	Kurarinone	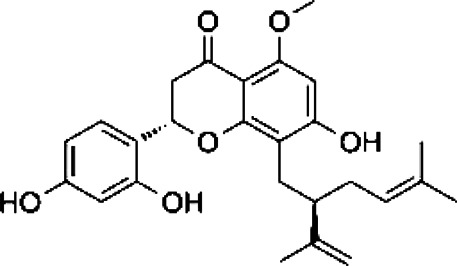	*Sophora flavescens*	PP-EAE	Th1	Inhibit	Xie et al. (2018)	
					Th17			
					Th2	Enhance		
	Naringenin	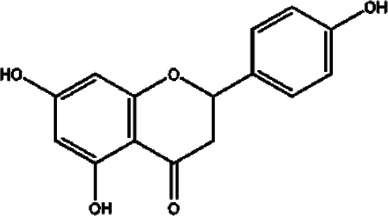	*Chrysanthemum Morifolium*	PP-EAE	Th1	Inhibit	Wang J. et al., (2018), Liu T. et al. (2020)	
					Th17			
					Th9			
	Eriodictyol	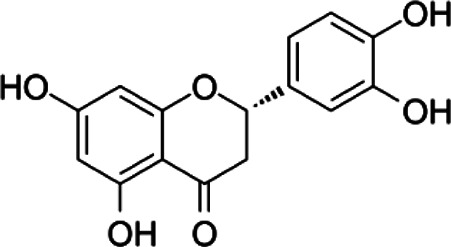	*Camellia sinensis*	PP-EAE	Th17	Inhibit	Lin et al. (2020), Yang T. et al. (2020)	
Glycoside	Astragaloside IV	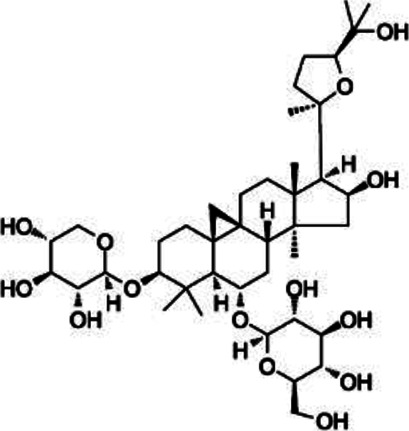	*Astragalus membranaceus*	PP-EAE	DC	Inhibit	Yang L. et al. (2020)	
	Paeoniflorin	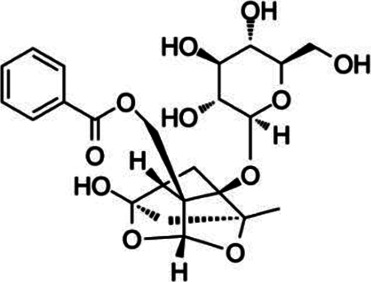	*Paeonia lactiflora*	PP-EAE	DC	Inhibit	Zhang et al. (2017)	
					Th17			
	Anemoside A3	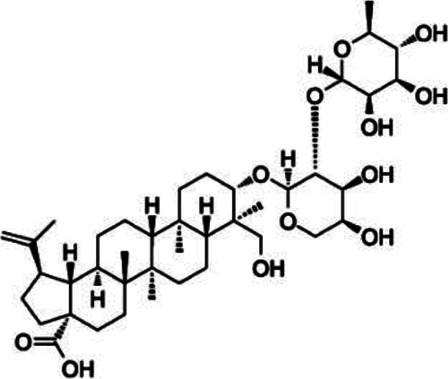	*Pulsatilla chinensis*	PP-EAE	Th1	Inhibit	Haghmorad et al. (2017)	
					Th17			
Triterpenoid	Ursolic acid	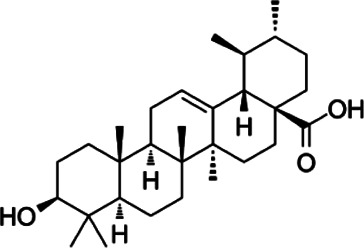	*Gardenia Jasminoides*	PP-EAE	OPC	Enhance	Sun Q. et al. (2020), Zhang Y. et al. (2020)	
					Th17	Inhibit		
	Carnosol	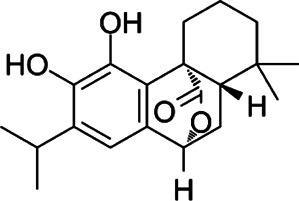	*Rosmarinus officanalis*	PP-EAE	Th17	Inhibit	Li X. et al. (2018)	
					Microglia			
	Glycyrrhizic acid	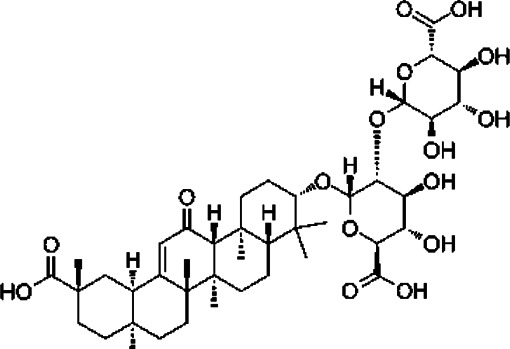	*Glycyrrhiza uralensis*	PP-EAE	OPC	Enhance	Zhou et al. (2015)	
					Microglia	Inhibit		
Others	Matrine	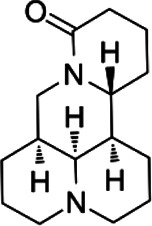	*Sophora flavescens*	PP-EAE	Astrogliosis	Inhibit	Moreno et al. (2013)	
	Scopoletin	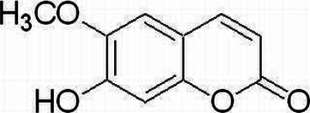	*Erycybe obtusifolia*	PP-EAE	DC	Inhibit	Zhang et al. (2019b)	
					Th1			
					Th17			
	6-Gingerol	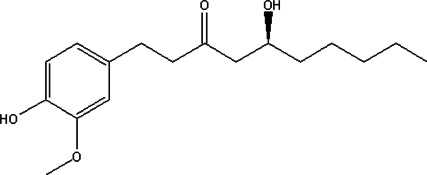	*Zingiber officanale*	PP-EAE	DC	Inhibit	Han et al. (2019)	
Others	Ellagic acid	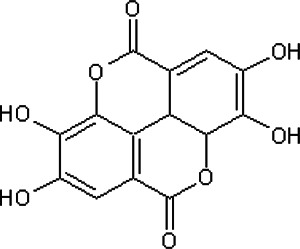	*Punica Grantum L.; Fragria Ananassa Duch*	PP-EAE	Astrogliosis microglia	Inhibit	Kiasalari et al. (2021)	

P-EAE represents MOG_35–55_ peptide-induced primary progressive EAE, and RR-EAE represents PLP_139–151_ peptide-induced relapsing-remitting EAE. This table lists the structure, source, animal model, and target cells of some Chinese herbal monomers. Based on the PP-EAE model, flavonoids mainly target inflammatory cells Th1 and Th17. While glycosides astragaloside IV and paeoniflorin target DC cells. Triterpenoid glycyrrhizic acid and ursolic acid could target OPC cells.

It is also worth noting that there is great significance in the elucidation of the molecular mechanisms of TCM monomers. However, in performing research on the mechanism of TCM in the treatment of diseases, there are many components in each type of Chinese medicine, and because of this complexity, coupled with the existence of multiple targets, the identification of practical components is a lengthy process. Thus, network pharmacology, molecular docking, and high-throughput screening, with targeting and timeliness, can be applied to the study of TCM and used for the treatment of EAE. All of these methods can be used to identify TCM monomers and play an essential role in the elucidation of how greater effectiveness in EAE treatment can be obtained from the molecular mechanisms of TCM.
